# Healthcare professionals' communication practices and preparedness in organised human papillomavirus-based cervical Cancer screening: A cross-sectional study in Central Portugal

**DOI:** 10.1016/j.pmedr.2026.103666

**Published:** 2026-10-10

**Authors:** Rita Sousa, José Alberto Fonseca-Moutinho, Fábio Gomes, Fernanda Loureiro, Ana Rita Goes, Patrícia Soares

**Affiliations:** a NOVA National School of Public Health, Public Health Research Centre, Comprehensive Health Research Center, CHRC, REAL, CCAL, NOVA University Lisbon, Av. Padre Cruz, 1600-560 Lisbon, Portugal; b Portuguese Institute of Oncology of Coimbra Francisco Gentil, Av. Bissaya Barreto 98, 3000-075 Coimbra, Portugal; c Health Sciences School, University of Beira Interior, Rua Marquês D'Ávila e Bolama, 6201–001 Covilhã, Portugal; d Public Health Department, Central Region Health Administration, Av. Dom Afonso Henriques, 141, 3001–553 Coimbra, Portugal; e National Institute of Health, Doutor Ricardo Jorge, Av. Padre Cruz, 11, 1649–016 Lisboa, Portugal

**Keywords:** Cervical cancer screening, Human papillomavirus, Health communication, Health personnel, Primary health care, Surveys and questionnaires

## Abstract

**Objective:**

To evaluate healthcare professionals' knowledge, attitudes, communication confidence and practices within an organised human papillomavirus (HPV)-based cervical cancer screening programme and identify factors associated with communication practices.

**Methods:**

A cross-sectional survey was conducted among 470 general practitioners and nurses involved in organised cervical cancer screening in Portugal's Central Region, January–March 2024. Participants completed a 28-item questionnaire. Exploratory factor analysis identified communication domains, and generalised linear models examined factors associated with communication practices.

**Results:**

Knowledge of HPV infection and screening was relatively high, with gaps in HPV test interpretation and clinical management. General practitioners reported greater communication confidence than nurses. HPV infection- and screening-related information was communicated more frequently than information concerning HPV transmission and oncogenic risk. Professional group, knowledge of HPV infection and screening guidelines, communication confidence and communication of HPV test results were independently associated with the HPV infection and screening-related communication domain. No significant associations were identified for communication of HPV transmission and oncogenic risk.

**Conclusions:**

Despite relatively high knowledge, gaps remained in knowledge and communication practices, particularly regarding HPV transmission and oncogenic risk. Professional training integrating clinical knowledge with communication about HPV, cervical cancer screening and cancer risk may strengthen healthcare professionals' preparedness for HPV-based screening.

## Highlights

• Communication gaps persisted despite relatively high HPV-related knowledge.
• General practitioners reported greater communication confidence than nurses.
• Knowledge and confidence were linked to more frequent communication.
• Communication was less frequent for HPV transmission and oncogenic risk.

## 1 Introduction

Although cervical cancer is largely preventable through human papillomavirus (HPV) vaccination and effective screening, it remains a major public health challenge worldwide (Bray et al., 2024). In response, the World Health Organization (WHO) has prioritised cervical cancer elimination through high HPV vaccination coverage, high-performance screening and timely access to diagnosis and treatment (WHO, 2020). Within this strategy, primary HPV testing has become the preferred screening approach because of its greater sensitivity for detecting cervical precancer and identifying women at increased risk of cervical cancer compared with cytology-based screening (Kyrgiou et al., 2020; World Health Organisation, 2021). The transition to primary HPV-based screening represents more than a technological advance; it has transformed cervical cancer prevention towards a more risk-based approach, incorporating HPV genotyping, extended screening intervals and self-sampling, with future screening pathways expected to become progressively tailored to individual risk profiles, including vaccination status (World Health Organization, 2026). As screening pathways become more complex, healthcare professionals play a pivotal role in providing clear, accurate and patient-centred communication to support informed decision-making throughout the screening process.

Portugal's Central Region has operated an organised cervical cancer screening programme since 1990, initially based on conventional cytology and fully transitioning to primary HPV screening in 2019 (Dinis et al., 2021; Dinis et al., 2023). Primary HPV screening requires healthcare professionals not only to implement screening protocols but also to explain HPV testing, communicate screening results and their implications, address misconceptions and support informed decision-making (Lin et al., 2015; Tatar et al., 2020). As screening pathways continue to evolve and incorporate new strategies such as HPV self-sampling, effective communication tailored to women's information needs will become increasingly important for informed participation in screening (World Health Organization, 2026; Gezels et al., 2025).

However, communicating HPV-related information effectively remains challenging because of HPV's association with sexual transmission, persistent misconceptions regarding HPV transmission and cancer risk, and the increasing complexity of HPV-based screening pathways (World Health Organization, 2026; Tatar et al., 2020; Marlow et al., 2020). Healthcare professionals report difficulties communicating HPV test results and their implications, while women may experience uncertainty and anxiety following HPV-positive results, particularly when communication is unclear or insufficient (Patel et al., 2017; Kiley et al., 2026; Patel et al., 2018). Although healthcare professionals' knowledge, attitudes and practices related to HPV testing have been extensively studied, with gaps identified in knowledge and adherence to screening recommendations (Tatar et al., 2020), their preparedness to communicate HPV-related information within organised primary HPV-based screening programmes has received less attention, particularly in routine primary care settings.

This study aimed to assess healthcare professionals' knowledge, attitudes, communication confidence and communication practices within an organised primary HPV-based cervical cancer screening programme, identify factors associated with communication practices, and characterise communication gaps that may inform future educational interventions and support programme implementation.

## 2 Methods

### 2.1 Study design and population

A cross-sectional study was conducted between 2 January and 31 March 2024 among primary healthcare professionals, including general practitioners and nurses, involved in the organised cervical cancer screening programme. The study was conducted across all primary care units under the Central Region Health Administration, comprising 86 primary healthcare centres with 175 health units (ARS Centro, 2026). These units employ approximately 2701 healthcare professionals, of whom 1834 are directly involved in the cervical cancer screening programme, and provide care to approximately 854,000 women, around half of whom are eligible for organised screening (ARS Centro, 2026).

During the study period, the organised screening pathway was based on primary HPV testing, with referral to colposcopy units determined by HPV genotype and reflex cytology results, as defined by the national screening framework (da República, 2017).

At the time of the study, no standardised procedure was established within the screening programme for communicating HPV test results to women, and communication practices varied across healthcare professionals and primary care units.

Healthcare professionals were involved in the screening programme as part of their routine primary care activities, according to the organization and allocation of tasks within each healthcare unit. Previous education or training related to cervical cancer, cervical cancer screening, HPV infection and screening sample collection were assessed as part of the questionnaire.

Eligible participants were required to have at least six months of experience in the organised cervical cancer screening programme. They were invited to complete a 28-item anonymous online questionnaire distributed through institutional email lists and social media (Facebook® and WhatsApp®).

### 2.2 Measures

Data were collected using a self-administered 28-item questionnaire developed from previously validated instruments (Lin et al., 2015; Patel et al., 2017; Caglioti et al., 2017; Hahn et al., 2021; Kwan et al., 2012; Liebermann et al., 2021; Nessler et al., 2019; Obol et al., 2021; Rodriguez et al., 2023; Roland et al., 2015; Sherman et al., 2018; Stormo et al., 2014; Tatar et al., 2018; Townsend et al., 2014; Waller et al., 2013) and a comprehensive literature review (da República, 2017; Chatzistamatiou et al., 2018; IARC, 2005; Schiffman and Wentzensen, 2013).

The questionnaire comprised four domains: 1) demographic and professional characteristics (age, gender, previous training, and years of experience in cervical cancer screening), 2) attitudes towards cervical cancer screening (perceptions of its importance, effectiveness and implementation), 3) knowledge of HPV infection, HPV testing, and screening guidelines (including HPV transmission, cervical cancer risk, HPV testing and screening recommendations), and 4) communication confidence and practices, including confidence in discussing HPV-related topics and the information communicated to women participating in screening.

The questionnaire followed the Knowledge, Attitudes and Practices framework (Andrade et al., 2020). Attitudes and perceptions were assessed using five-point Likert scales, whereas knowledge was evaluated using multiple-choice and true/false questions. Three clinical vignettes simulating routine clinical scenarios were used to assess knowledge of clinical management and communication practices (Townsend et al., 2014). Participants selected the appropriate clinical management and indicated how frequently they would communicate specific HPV- and screening-related information when discussing test results with patients. Communication items were rated as *always*, *most of the time, sometimes or never,* with an additional *no need to communicate the result* option in the first two clinical vignettes. Communication practices were therefore assessed through the self-reported frequency with which participants would communicate specific information in the clinical scenarios. These items were subsequently grouped through exploratory factor analysis into distinct communication domains, reflecting the content addressed in communication rather than an overall measure of communication quality. An open-ended question allowed participants to provide additional comments.

Content validity was assessed by an expert panel, following established methodological guidance (Andrade et al., 2020). The panel comprised seven healthcare professionals: two primary care professionals (one general practitioner and one nurse), two public health specialists, two colposcopy specialists (one gynaecologist and one nurse), and one cytopathologist. Experts independently assessed item relevance, clarity, comprehension, response options and feasibility using a structured validation form (*Supplementary Material 1.1*). Following the initial review, the questionnaire was revised based on expert feedback and subsequently reassessed by the panel, with further modifications made where appropriate.

The questionnaire was pretested with 20 healthcare professionals from three Portuguese regions: the Northern Region (five participants), Algarve Region (six participants), and Central Region (nine participants). Subsequently, a focus group in the Central Region assessed item clarity and technical functionality, including questionnaire navigation and response selection. Minor revisions were made prior to implementation.

The final questionnaire was administered using the Research Electronic Data Capture (REDCap®) platform (*Supplementary Material* 1.2).

### 2.3 Statistical analysis

Descriptive statistics were used to summarise participants' demographic and professional characteristics. Three knowledge scores were calculated from the number of correct responses within each knowledge domain and expressed as percentages. Five-point Likert items were analysed as continuous variables. Negatively worded items were reverse-coded before analysis so that higher scores consistently represented higher levels of the construct being assessed. Group comparisons were performed using independent-samples *t-*tests or Mann–Whitney *U* tests, as appropriate, and chi-square tests for categorical variables. Statistical significance was defined as *p* < 0.05.

Exploratory factor analysis (EFA) was used to identify underlying domains among questionnaire items. Sampling adequacy was assessed using the Kaiser–Meyer–Olkin measure and Bartlett's test of sphericity. Principal component analysis was used as the extraction method, followed by Varimax rotation and Kaiser normalisation. Components with eigenvalues >1 were retained. Internal consistency of the identified domains was assessed using Cronbach's alpha.

EFA was conducted for awareness and perceptions regarding cervical cancer screening and HPV testing, communication confidence, communication of HPV test results, and communication practices. Factor scores derived from these analyses were used in subsequent analyses. Three domains of awareness and perceptions regarding cervical cancer screening and HPV testing were identified: perceived logistical barriers, awareness of screening effectiveness, and perceived safety and reliability of HPV testing (Supplementary Table 1). Communication practice comprised two domains: *HPV infection and screening-related information* and *HPV transmission and oncogenic risk* (Table 1)*.* The factor scores representing these two domains were used as the outcome variables in the regression analyses, with higher scores indicating more frequent self-reported communication of the information represented within each domain.

**Table 1:** Exploratory factor analysis of healthcare professionals' communication practices regarding human papillomavirus infection and cervical cancer screening in Central Portugal, 2024.

	Factor 1 Human papillomavirus infection and screening-related information	Factor 2 Human papillomavirus transmission and oncogenic risk
**Item**		
Most human papillomavirus infections are transient and do not progress to cervical precancer or cancer	0.81	
Human papillomavirus infection is usually asymptomatic	0.81	
Human papillomavirus infection may persist for many years, making it difficult to determine when it was acquired	0.78	
Most human papillomavirus infections clear spontaneously without treatment, particularly in young women	0.72	
Human papillomavirus vaccination protects against the most oncogenic types but does not prevent infection with all human papillomavirus types	0.70	
Primary human papillomavirus testing is recommended because it is more sensitive than cytology	0.69	
Although human papillomavirus infection may be transient, human papillomavirus type 16 infection should not be underestimated because of its higher oncogenic risk	0.62	
Smoking increases the risk of progression to cervical precancer and cancer	0.60	
Human papillomavirus is sexually transmitted (Vignette 1)		0.87
Human papillomavirus is sexually transmitted (Vignette 2)		0.87
Human papillomavirus is a virus that can cause cancer (Vignette 1)		0.86
Human papillomavirus is a virus that can cause cancer (Vignette 2)		0.85
Explained variance	34.5%	24.9%
Eigenvalue	4.17	2.95
Internal consistency (Cronbach's α)	0.853	0.882

**Extraction method:** principal component analysis**; rotation method**: varimax with Kaiser normalisation. **Total variance explained**: 59.4%.

Generalised linear models with a normal distribution, identity link function and robust standard errors were used to identify factors associated with each communication practice domain. Variables statistically significantly associated with each outcome in the univariable analysis were included in the corresponding multivariable model. Results are presented as regression coefficients (B) with 95% confidence intervals (CIs).

All analyses were performed using IBM® *SPSS*® *Statistics* software, version 29.0.1.0.

### 2.4 Ethical approval and consent to participate

Ethical approval was obtained from the Ethics Committee of the Regional Health Administration Centre of Portugal (CES.116/2022; 19 January 2023) and from the Ethics Committee of the Portuguese Institute of Oncology of Coimbra Francisco Gentil (TI22/2022; 20 October 2022). The study was conducted in accordance with the Declaration of Helsinki and institutional guidelines for the protection, privacy and confidentiality of research participants. All participants provided informed consent before beginning the survey as described in *Supplementary Material 1.2.* Data were collected anonymously.

## 3 Results

A total of 725 healthcare professionals initiated the survey. After excluding 255 participants (35.2%) because of incomplete questionnaires or employment outside the Central Region Health Administration area, the final sample comprised 470 participants, representing 25.6% of the 1834 healthcare professionals involved in the organised cervical cancer screening programme in Portugal's Central Region. Of these, 227 (48.3%) were general practitioners and 243 (51.7%) were nurses (Table 2).

**Table 2:** Demographic and professional characteristics of healthcare professionals participating in organised cervical cancer screening in Central Portugal, 2024.

Characteristic	Total (*n* = 470)	General practitioners (*n* = 227)	Nurses (*n* = 243)
Gender, n (%)			
Female	408 (86.8%)	182 (80.2%)	226 (93.0%)
Male	62 (13.2%)	45 (19.8%)	17 (7.0%)
Age, years, mean (range)	44.8 (25–70)	40.5 (25–70)	48.8 (25–65)
Age group, n (%)			
<30	41 (8.7%)	40 (17.6%)	1 (0.4%)
30–49	277 (58.9%)	145 (63.9%)	132 (54.3%)
≥50	152 (32.3%)	42 (18.5%)	110 (45.3%)
Practice setting, n (%)			
Urban	373 (79.4%)	194 (85.5%)	179 (73.7%)
Rural	97 (20.6%)	33 (14.5%)	64 (26.3%)
Years in practice, n (%)			
<1	11 (2.3%)	8 (3.5%)	3 (1.2%)
1–4	57 (12.1%)	42 (18.5%)	15 (6.2%)
5–9	93 (19.8%)	59 (26.0%)	34 (14.0%)
≥10	309 (65.7%)	118 (52.0%)	191 (78.6%)
Time spent with cervical cancer screening per week, n (%)			
<2 h	185 (39.4%)	80 (35.2%)	105 (43.2%)
2–4 h	267 (56.8%)	144 (63.4%)	123 (50.6%)
≥5 h	11 (2.3%)	2 (0.9%)	9 (3.7%)
Prefer not to respond	7 (1.5%)	1 (0.4%)	6 (2.5%)
Previous education or training related to cervical cancer screening, n (%)			
Human papillomavirus infection			
Yes	197 (41.9%)	102 (44.9%)	95 (39.1%)
No	270 (57.4%)	123 (54.2%)	147 (60.5%)
Prefer not to respond	3 (0.6%)	2 (0.9%)	1 (0.4%)
Cervical cancer screening programme			
Yes	239 (50.9%)	133 (58.6%)	106 (43.6%)
No	230 (48.9%)	94 (41.4%)	136 (56.0%)
Prefer not to respond	1 (0.2%)	0	1 (0.4%)
Cervical sampling			
Yes	222 (47.2%)	125 (55.1%)	97 (39.9%)
No	247 (52.6%)	102 (44.9%)	145 (59.7%)
Prefer not to say	1 (0.2%)	0	1 (0.4%)

CCS, cervical cancer screening.

Compared with general practitioners, nurses were generally older and more likely to work in rural settings, whereas general practitioners more frequently reported previous education or training across the assessed screening-related areas. Most participants had at least 10 years of professional experience (65.7%), and 56.8% reported spending 2–4 h per week on cervical cancer screening, more frequently among general practitioners than nurses (63.4% vs. 50.6%) (Table 2).

Overall, healthcare professionals demonstrated relatively high knowledge of HPV infection and cervical cancer screening. Knowledge scores were highest for HPV infection, followed by cervical cancer screening guidelines, whereas scores for HPV testing were comparatively lower. General practitioners had higher mean scores across all three knowledge domains. Knowledge gaps were identified in HPV transmissibility, interpretation of HPV test results, and clinical management following screening results, with only 50.9% correctly recognising that HPV is not exclusively sexually transmitted (Supplementary Table 2).

Awareness of the benefits of cervical cancer screening was high in both professional groups, whereas perceptions regarding HPV testing were less consistent, with general practitioners showing greater awareness than nurses of test sensitivity and the recommended screening interval following a negative test (Supplementary Table 3).

Most participants considered cervical cancer screening highly important and reported routinely recommending screening to eligible women (Fig. 1, Fig. 2). Invitations were most commonly made during family planning visits and other routine consultations, although nurses reported a broader range of invitation strategies.

**Fig. 1:**
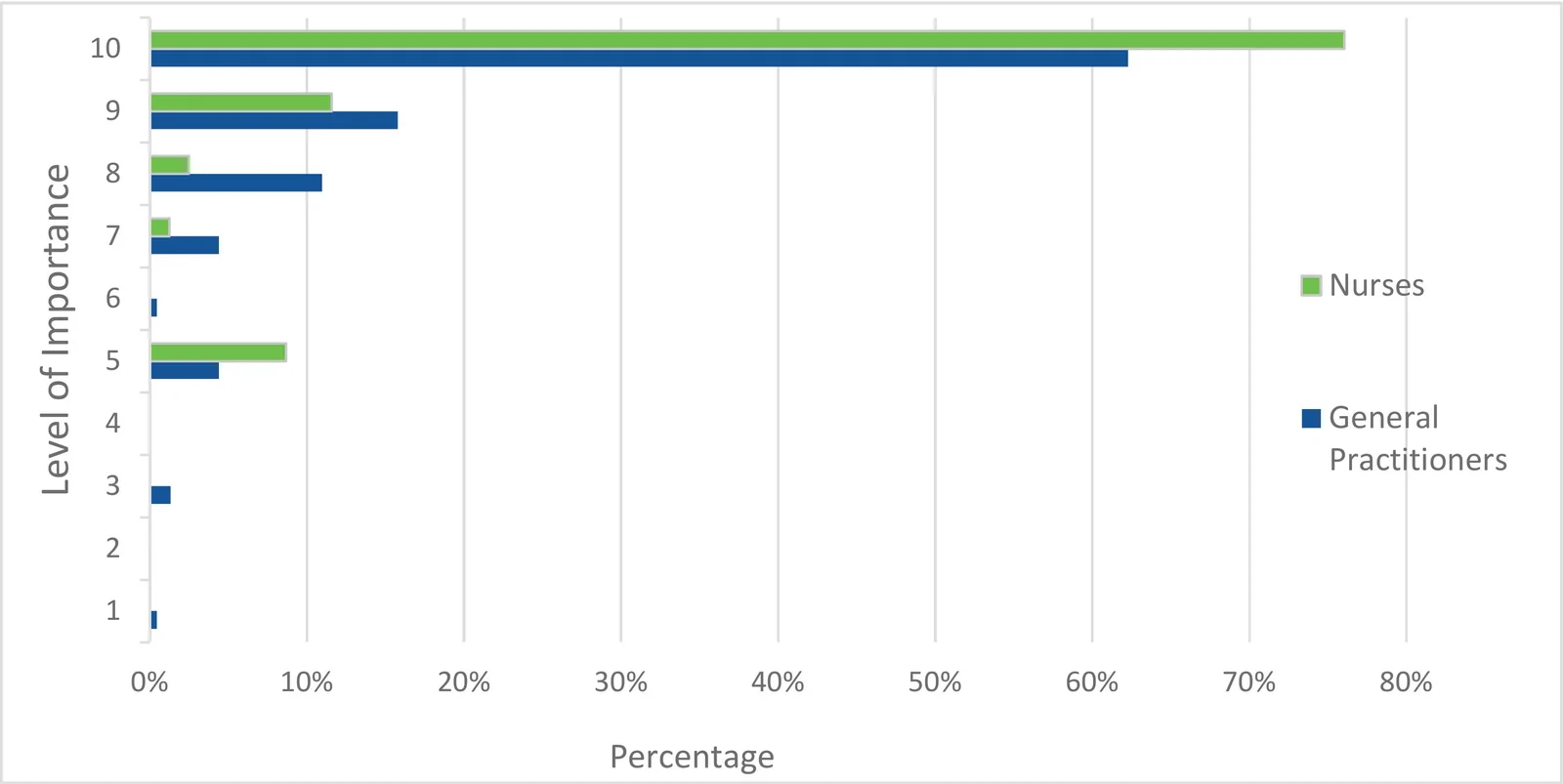
Perceived importance of cervical cancer screening among general practitioners and nurses in Central Portugal, 2024. Ratings were based on a 10-point scale (1 = least important; 10 = most important).

**Fig. 2:**
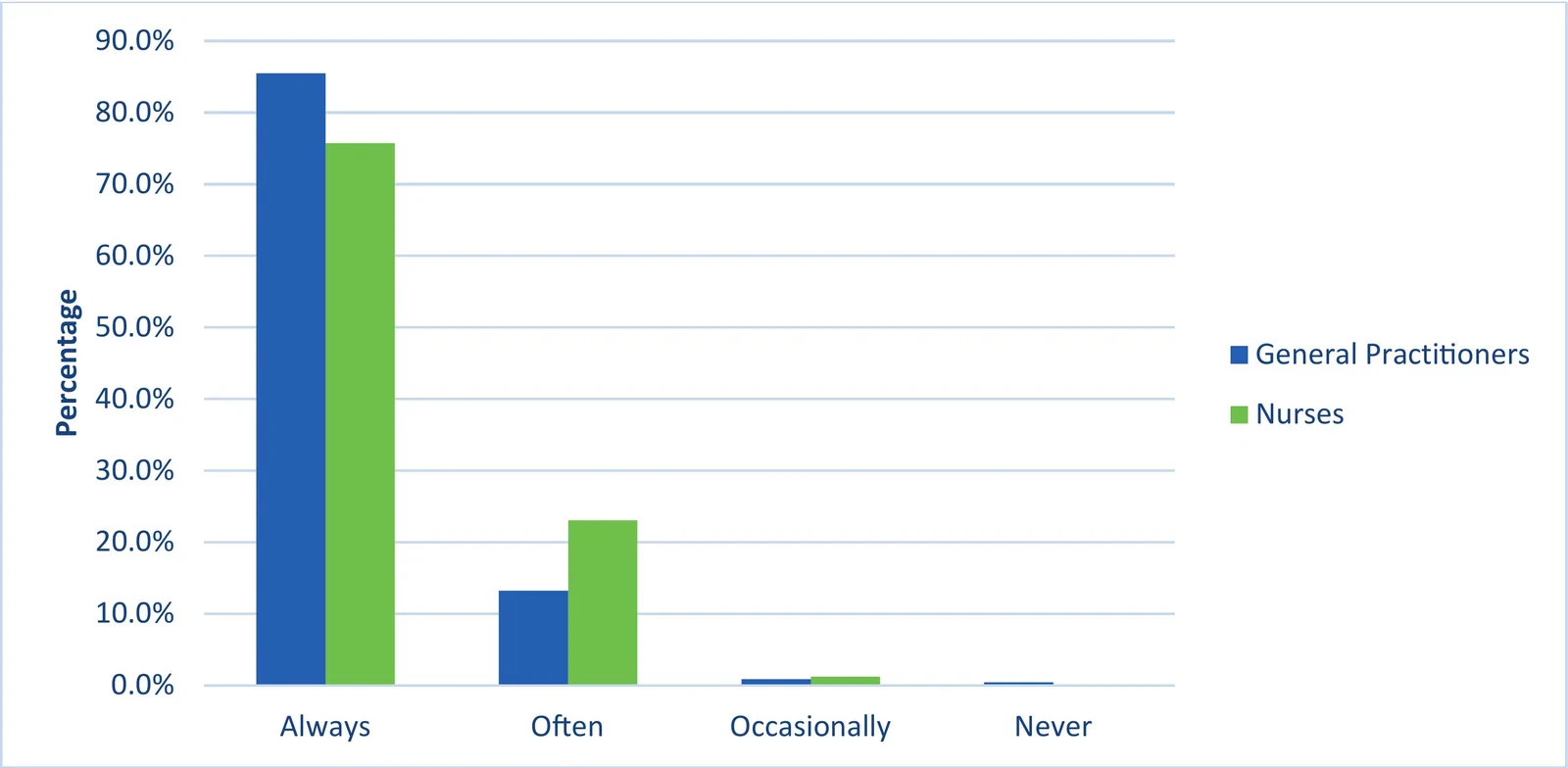
Frequency of healthcare professionals' recommendations for cervical cancer screening among eligible women in Central Portugal, 2024.

Perceptions of the transition to primary HPV testing were generally favourable but highlighted organisational challenges. Few participants considered that the transition had increased the time spent on screening or reduced screening adherence among patients or healthcare professionals. However, general practitioners were more likely than nurses to perceive delays in scheduling first appointments at colposcopy units as a barrier to effective screening (39.6% vs. 26.7%, *p* = 0.02) (Supplementary Table 4).

Overall confidence in addressing HPV-related topics was high in both professional groups, although general practitioners reported greater confidence than nurses across all assessed topics. General practitioners also reported more frequent communication of positive HPV test results, whereas communication of negative results was infrequent in both professional groups (Fig. 3; Supplementary Tables 5 and 6).

**Fig. 3:**
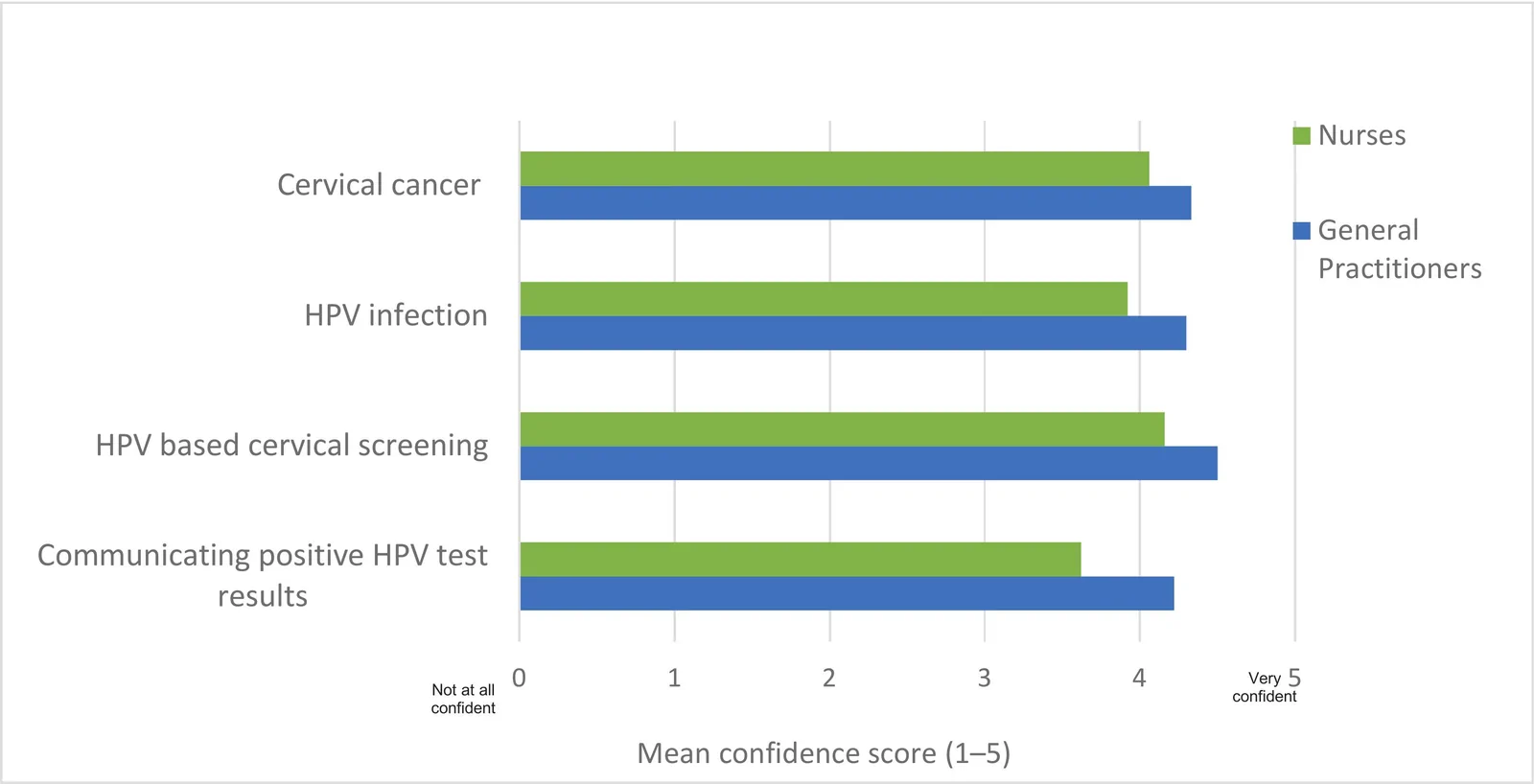
Mean confidence scores for addressing human papillomavirus-related topics among general practitioners and nurses, Central Portugal, 2024. Higher scores indicate greater confidence in addressing HPV-related topics.

Across the three clinical vignettes, healthcare professionals reported more frequent communication of information within the HPV infection and screening-related domain than information concerning HPV transmission and oncogenic risk. Sexual transmission and cancer risk were among the least frequently communicated messages, with nurses generally reporting lower frequencies than general practitioners (Supplementary Tables 5 and 6).

In the multivariable regression analysis, professional group, knowledge of HPV infection and cervical cancer screening guidelines, communication confidence, and communication of HPV test results were independently associated with the HPV infection and screening-related communication domain, with general practitioners reporting higher scores than nurses. For communication of human papillomavirus transmission and oncogenic risk, professional group showed a borderline association in the univariable analysis, but no variables met the significance criterion for inclusion in a multivariable model (Table 3).

**Table 3:** Factors associated with healthcare professionals' communication practices regarding human papillomavirus infection and cervical cancer screening in Central Portugal, 2024.

	Communication of human papillomavirus infection and screening-related information		Communication of human papillomavirus transmission and oncogenic risk
	**Crude B *(95% confidence interval)***	**Adjusted B *(95% confidence interval)***	**Crude B *(95% confidence interval)***
Demographic characteristics			
Age (years)	−0.02 (−0.03, −0.01)	−0.01 (−0.02, −0.001)	0.01 (−0.003, 0.01)
Gender			
Male (reference)	–	–	–
Female	0.05 (−0.26, 0.35)	–	−0.01 (−0.30, 0.28)
Professional characteristics			
Professional group			
Nurses (reference)	–	–	–
General practitioners	0.54 (0.36, 0.71)	0.19 (0.01, 0.37)	−0.18 (−0.37, 0.003)
Practice setting			
Urban (reference)	–	–	–
Rural	0.09 (−0.11, 0.29)	–	−0.09 (−0.29, 0.11)
Previous education or training related to cervical cancer screening			
Yes (reference)	–	–	–
No	−0.20 (−0.39, 0.01)	−0.03 (−0.19, 0.13)	−0.01 (−0.19, 0.18)
Knowledge			
Human papillomavirus infection	0.25 (0.13, 0.36)	0.14 (0.05, 0.23)	0.07 (−0.04, 0.18)
Human papillomavirus test	0.30 (0.15, 0.46)	0.09 (−0.04, 0.22)	−0.08 (−0.22, 0.07)
Cervical cancer screening guidelines	0.38 (0.24, 0.52)	0.24 (0.12, 0.35)	−0.05 (−0.19, 0.09)
Awareness, perceptions and barriers (factor scores)			
Perceived logistical barriers	0.13 (0.01, 0.25)	0.06 (−0.03, 0.15)	−0.01 (−0.12, 0.09)
Awareness of screening effectiveness	<0.01 (−0.10, 0.10)	–	<0.01 (−0.12, 0.11)
Perceived safety and reliability of screening tests	0.14 (0.03, 0.25)	−0.01 (−0.11, 0.09)	−0.02 (−0.13, 0.08)
Communication-related variables			
Confidence in addressing human papillomavirus-related topics	0.30 (0.17, 0.42)	0.11 (0.01, 0.21)	0.06 (−0.05, 0.16)
Communication of human papillomavirus test results	0.33 (0.20, 0.46)	0.25 (0.15, 0.36)	0.07 (−0.05, 0.18)

B, regression coefficient. Factor scores were derived from the exploratory factor analysis. No multivariable model was fitted for communication on human papillomavirus transmission and oncogenic risk because no variables were statistically significant in the univariable analyses.

## 4 Discussion

This study showed that, despite relatively high knowledge of HPV infection and cervical cancer screening, important communication gaps remain among healthcare professionals participating in an organised HPV-based screening programme. These gaps were particularly evident in the communication of HPV transmission and oncogenic risk, indicating that knowledge alone does not fully account for differences in communication practices. By simultaneously evaluating knowledge, attitudes, communication confidence and communication practices, this study provides a broader understanding of healthcare professionals' preparedness to support organised HPV-based cervical cancer screening.

Important knowledge gaps remained regarding HPV test interpretation and clinical management following abnormal screening results. These findings are consistent with previous evidence indicating that the transition from cytology- to HPV-based screening has increased the complexity of clinical decision-making, requiring healthcare professionals to understand updated screening algorithms and their practical application in routine care (World Health Organization, 2026; Tatar et al., 2020; Roland et al., 2015). The persistence of knowledge gaps despite widespread implementation of HPV-based screening suggests that educational strategies should extend beyond guideline dissemination and place greater emphasis on HPV test interpretation, risk-based management and clinical decision-making.

Communication confidence also differed according to professional group. General practitioners consistently reported greater confidence than nurses across all HPV-related topics, particularly when discussing positive HPV test results. These differences may reflect variations in professional training and clinical responsibilities. This finding is consistent with previous evidence linking healthcare professionals' HPV knowledge and preparedness with confidence in responding to patients' questions (Patel et al., 2017; Sherman et al., 2018). The independent association between communication confidence and more frequent communication of HPV infection- and screening-related information further suggests that confidence may be relevant to the extent to which healthcare professionals address this information.

One of the most relevant findings was the difference between communication domains. Across the clinical vignettes, HPV infection- and screening-related information was communicated more consistently than information concerning HPV transmission and oncogenic risk. Messages concerning sexual transmission and cancer risk were among those communicated less consistently, particularly among nurses. This distinction is particularly relevant because the sexually transmitted nature of HPV and the implications of a positive result may generate anxiety, embarrassment and concerns about stigma, particularly when women have limited information about HPV (Patel et al., 2018). More recent evidence has also identified persistent information and communication needs following HPV-positive results (Kiley et al., 2026).

The multivariable analysis showed that professional group, knowledge of HPV infection and cervical cancer screening guidelines, communication confidence, and communication of HPV test results were independently associated with the HPV infection and screening-related communication domain, with general practitioners reporting higher scores than nurses. By contrast, no significant associations were identified for communication of HPV transmission and oncogenic risk. This suggests that communication within this domain may be influenced by factors not captured in the present study. Previous research has similarly highlighted challenges in communicating HPV-related information and the implications of HPV test results (Lin et al., 2015). Together, these findings reinforce the importance of communication as part of the implementation of HPV-based screening.

From a programme implementation perspective, these findings highlight the importance of healthcare professional preparedness as HPV-based screening continues to evolve. As screening moves towards risk-based approaches, HPV self-sampling and more personalised screening pathways (World Health Organization, 2026), healthcare professionals will need to communicate increasingly complex and potentially sensitive information. Professional training should therefore combine technical updates with communication training focused on HPV result interpretation, communication of screening results, and discussion of HPV transmission and oncogenic risk. Previous research has also highlighted the psychological and emotional impact of HPV-positive results, including anxiety, embarrassment and concerns about stigma (Kiley et al., 2026; Patel et al., 2018), although these dimensions were not directly assessed in the present study. Such training may support women's understanding and informed decision-making as organised HPV-based cervical cancer screening continues to evolve.

The strengths of this study include a substantial sample of healthcare professionals involved in an organised cervical cancer screening programme and a comprehensive assessment of knowledge, attitudes, communication confidence and communication practices. The combination of exploratory factor analysis and multivariable regression modelling also enabled the identification of distinct communication domains and factors independently associated with communication practices.

Several limitations should be acknowledged. The cross-sectional design precludes causal inference regarding the associations identified. Communication practices were self-reported and may therefore be subject to social desirability bias. Although clinical vignettes provide a useful approximation of routine practice, they cannot fully reflect the complexity of real clinical encounters, including qualitative aspects such as tone, empathy, emotional responsiveness, non-verbal communication and contextual influences. The study assessed the frequency with which specific HPV- and screening-related information would be communicated, rather than the quality of communication or its psychosocial dimensions. Stigma and emotional concerns associated with HPV-positive results were not directly assessed and therefore cannot be evaluated from the present findings. In addition, the study was conducted within a single regional organised screening programme, which may limit the generalisability of the findings to healthcare systems with different screening pathways or professional roles.

Future research should evaluate whether communication-focused educational interventions improve healthcare professionals' communication practices and women's understanding, experience and participation in organised cervical cancer screening.

## 5 Conclusion

Healthcare professionals participating in an organised HPV-based cervical cancer screening programme demonstrated relatively high knowledge of HPV infection and screening recommendations, but important gaps remained in both knowledge and communication practices, particularly regarding HPV transmission and oncogenic risk. These findings support professional training that integrates clinical knowledge with communication about HPV, cervical cancer screening and cancer risk. As organised cervical cancer screening evolves towards increasingly risk-based and personalised pathways, strengthening healthcare professionals' knowledge and communication preparedness may support informed participation and effective programme implementation.

## CRediT authorship contribution statement

**Rita Sousa:** Writing – review & editing, Writing – original draft, Validation, Project administration, Methodology, Investigation, Data curation, Conceptualization. **José Alberto Fonseca-Moutinho:** Writing – review & editing, Validation, Supervision, Conceptualization. **Fábio Gomes:** Writing – review & editing, Conceptualization. **Fernanda Loureiro:** Writing – review & editing, Conceptualization. **Ana Rita Goes:** Writing – review & editing, Validation, Supervision, Methodology, Conceptualization. **Patrícia Soares:** Writing – review & editing, Validation, Supervision, Methodology, Conceptualization.

## Informed consent statement

Informed consent was obtained from all subjects involved in the study, as described in Supplementary Material 1.2. Collected data were anonymised to ensure participant confidentiality and data protection.

## Institutional review board statement

The study was conducted in accordance with the Declaration of Helsinki and approved by the Ethics Committee of the Central Region Health Administration of Portugal (CES.116/2022; 19 January 2023) and the Ethics Committee of the Portuguese Institute of Oncology of Coimbra Francisco Gentil (TI22/2022; 20 October 2022).

## Funding

This work was partially supported by the 10.13039/501100001871Foundation for Science and Technology (reference: CEECINST/00049/2021/CP2817/CT0001).

## Declaration of competing interest

The authors declare that they have no known competing financial interests or personal relationships that could have appeared to influence the work reported in this paper.

## Data Availability

Data will be made available on request.
