# Supplementary material for: Healthcare professionals' communication practices and preparedness in organised human papillomavirus-based cervical Cancer screening: A cross-sectional study in Central Portugal
Source: Prev Med Rep. 2026 Oct 10;71:103666. doi: 10.1016/j.pmedr.2026.103666 (PMC13682059; doi:10.1016/j.pmedr.2026.103666)
Supplement: Supplementary file 1 [file mmc1.docx]

**Title:** Healthcare Professionals' Communication Practices and Preparedness in Organised Human Papillomavirus-Based Cervical Cancer Screening: A Cross-Sectional Study in Central Portugal

**Appendix A - Supplementary Materials**

**Supplementary material 1**

**1.1. Validation form:** Content validation criteria

Each expert received an Excel file containing all questionnaire items and was asked to evaluate them using the criteria below:

| Item | Options | Comment | Suggestions |
| --- | --- | --- | --- |
| Item relevance | 1. Acceptable 2. Unacceptable | 1. Keep 2. Modify |  |
| Text and question clarity | 1. Acceptable 2. Unacceptable | 1. Clear 2. Not clear |  |
| Text Comprehension | 1. Acceptable 2. Unacceptable | 1. Easy 2. Difficult |  |
| Answer choices/Scales | 1. Acceptable 2. Unacceptable | 1. Keep 2. Modify |  |
| Ease of questionnaire completion | 1. Acceptable 2. Unacceptable | 1. Easy 2. Not intuitive |  |
| Time to complete | 1. Acceptable 2. Unacceptable | 1. Reasonable 2. Excessive |  |
| Length of the questionnaire | 1. Acceptable 2. Unacceptable | 1. Reasonable 2. Excessive |  |
| Structure and layout | 1. Acceptable 2. Unacceptable | 3. Keep 4. Modify |  |

Additional feedback was collected through open-ended fields:

- Expert suggestions during content validation
- Primary care professionals’ suggestions during pre-testing
  (Note: Pre-testing was conducted after incorporating expert feedback.)

**1.2. Survey:**

Attitudes, knowledge and perceptions of Primary Health Care professionals towards Cervical Cancer Screening

Dear Sir/Madam,

You are invited to take part in a research study being conducted by Rita Sousa, a PhD candidate at NOVA National School of Public Health, NOVA University Lisbon, and a gynaecologist at the Portuguese Institute of Oncology of Coimbra (IPOC). This study is part of the research protocol for the doctorate thesis.

The aim of this study is to understand the practices and perspectives of primary healthcare providers regarding cervical cancer screening (CCS), with a focus on general practitioners and nurses who are currently involved in the program.

We will not ask you for your name, contact information, or any other information that could be used to identify you; the information gathered is anonymous and private.

Completing this questionnaire carries no risk, and you can stop at any moment. NOVA University Lisbon will only use the information gathered for academic and scientific purposes.

Since there are no right or incorrect answers, we kindly ask that you respond to all queries and concerns in a sincere and honest manner.

Response time is anticipated to be 15 minutes.

If you require any further assistance with this study, you can email Rita Sousa.

[rmr.fernando@ensp.unl.pt](mailto:rmr.fernando@ensp.unl.pt)

Do you consent to taking part in the study and granting permission for the collection and processing of the data obtained from this survey?

1. Yes
2. No

| Survey form | |
| --- | --- |
| Age |  |
| Gender | 1. Female 2. Male 3. Other 4. Prefer not to say |
| Health unit where you work | 1. CS Viseu I 2. UCSP Águeda I 3. UCSP Águeda II 4. UCSP Águeda III 5. UCSP Águeda V   (...)  175. USF Vitrius  176. Other |
| Professional Group | 1. General Practitioner 2. Nurse |
| How long have you been involved in cervical cancer screening (CCS)? | 1. < 1 year 2. 1-5 years 3. 5-9 years 4. ≥10 years |
| Have you received specific training in the following areas? | |
| cervical cancer | 1. Yes 2. No 3. Prefer not to say |
| cervical cancer screening | 1. Yes 2. No 3. Prefer not to say |
| HPV infection | 1. Yes 2. No 3. Prefer not to say |
| CCS sample collection | 1. Yes 2. No 3. Prefer not to say |
| How much time do you allocate to CCS on a weekly basis? | 1. < 2 hours 2. 2 - 4 hours 3. ≥ 5 hours 4. Prefer not to say |
| To what extent do you prioritize cervical cancer screening? | 0-Not important at all …….10-very important |
| Regarding CCS, select the option that you consider most appropriate | |
| CCS helps to reduce mortality from cervical cancer | 1. Strongly disagree 2. Disagree 3. Neither agree nor disagree 4. Agree 5. Strongly agree 6. Prefer not to respond |
| CCS allows the treatment of pre-invasive disease and prevents progression to cervical cancer | 1. Strongly disagree 2. Disagree 3. Neither agree nor disagree 4. Agree 5. Strongly agree 6. Prefer not to say |
| HPV test is more sensitive than cytology | 1. Strongly disagree 2. Disagree 3. Neither agree nor disagree 4. Agree 5. Strongly agree 6. Prefer not to say |
| With a negative HPV test is safe to repeat the CCS at five years | 1. Strongly disagree 2. Disagree 3. Neither agree nor disagree 4. Agree 5. Strongly agree 6. Prefer not to say |
| Compared with cytology, what level of confidence does HPV testing provide in the context of CCS? | 1. Higher confidence 2. Less confidence 3. The same confidence 4. Prefer not to say |
| What is the age at which the CCS should begin, based on current recommendations? | 1. 25 years 2. 30 years 3. 45 years old 4. 50 years |
| When should the HPV test be repeated after a negative result? | 1. Annual 2. 2/2 years 3. 3/3 years 4. 5/5 years |
| Do you think the method of taking cervicovaginal samples for HPV testing is: | 1. Very easy 2. Easy 3. Neither easy nor difficult 4. Difficult 5. Not applicable |
| Does participating in the screening interfere with your need to perform other caregiving duties? | 1. No 2. Neither yes nor no 3. Yes 4. Don't know/prefer not to say |
| Select the option you believe is most appropriate regarding the switch to the primary HPV test. | |
| - The transition to HPV testing has led to more time spent on screening | 1. Strongly disagree 2. Disagree 3. Neither agree nor disagree 4. Agree 5. Strongly agree 6. Prefer not to say |
| - The transition to HPV testing resulted in a decrease in HCPs’ compliance to screening program | 1. Strongly disagree 2. Disagree 3. Neither agree nor disagree 4. Agree 5. Strongly agree 6. Prefer not to say |
| - The transition to HPV testing resulted in a decrease in patients’ compliance to screening program | 1. Strongly disagree 2. Disagree 3. Neither agree nor disagree 4. Agree 5. Strongly agree 6. Prefer not to say |
| - The transition to HPV testing triggered an excessive referral to hospital units | 1. Strongly disagree 2. Disagree 3. Neither agree nor disagree 4. Agree 5. Strongly agree 6. Prefer not to say |
| - The delay in scheduling first appointments at colposcopy units’ delays is a barrier to effective screening. | 1. Strongly disagree 2. Disagree 3. Neither agree nor disagree 4. Agree 5. Strongly agree 6. Prefer not to say |
| Select the option you think is the most appropriate given the recommendation to perform the CCS. | |
| - Do you usually recommend screening for cervical cancer? | 1. Always 2. Often 3. Occasionally 4. Never 5. Prefer not to say |
| - How do you invite individuals for the screening test? | 1. In the family planning visit 2. During a standard clinical surveillance evaluation 3. Going to the health unit with a family member 4. By invitation letter 5. By phone 6. By email 7. By SMS 8. None of the above |
| What is your confidence level to address the following topics with your patients? | |
| - Cervical cancer | 1. Not confident 2. Somewhat confident 3. Indifferent 4. Confident 5. Very confident 6. Don't know/prefer not to say |
| - HPV infection | 1. Not confident 2. Somewhat confident 3. Indifferent 4. Confident 5. Very confident 6. Don't know/prefer not to say |
| - HPV-based CCS | 1. Not confident 2. Somewhat confident 3. Indifferent 4. Confident 5. Very confident 6. Don't know/prefer not to say |
| - Explaining a positive test result | 1. Not confident 2. Somewhat confident 3. Indifferent 4. Confident 5. Very confident 6. Don't know/prefer not to say |
| In relation to HPV infection, do you think that | |
| - HPV infection is rare. | 1. True 2. False 3. Don’t know |
| - HPV infection only occurs in individuals with multiple sexual partners. | 1. True 2. False 3. Don’t know |
| - HPV is exclusively sexually transmitted. | 1. True 2. False 3. Don’t know |
| - It is usually asymptomatic. | 1. True 2. False 3. Don’t know |
| - It is not possible to determine when HPV infection occurred. | 1. True 2. False 3. Don’t know |
| - Most sexually active individuals will acquire HPV at some point. | 1. True 2. False 3. Don’t know |
| - Persistent HPV infection is associated with pre-cancer and cancer. | 1. True 2. False 3. Don’t know |
| In relation to HPV test, do you think that | |
| - HPV testing effectively assesses cervical cancer risk. | 1. True 2. False 3. Don’t know |
| - A positive HPV test is always an indication for colposcopy. | 1. True 2. False 3. Don’t know |
| - A positive HPV test means the woman has or will develop cervical cancer. | 1. True 2. False 3. Don’t know |
| - A negative HPV test means a very low risk of cervical cancer | 1. True 2. False 3. Don’t know |
| Regarding the disclosure of HPV test results, how often do you inform: | |
| - Positive HPV test result | 1. Always 2. Often 3. Occasionally 4. Never 5. No need to notify 6. Prefer not to say |
| - Negative HPV test result | 1. Always 2. Often 3. Occasionally 4. Never 5. No need to notify 6. Prefer not to say |
| The following three vignettes contain a description of clinical scenarios with a positive HPV test result. Select the options you consider most appropriate in guiding and informing patients. | |
| Vignette 1 |  |
| 26-year-old patient, vaccinated against HPV with the quadrivalent vaccine, nulliparous, non-smoker. Invited to perform cervical cancer screening with HPV test during the family planning consultation. The test is "positive for high-risk HPV of other types than 16 and 18". Due to this result, reflex cytology was performed, whose result is "negative for intraepithelial lesion or malignancy - NILM". | |
| What is the correct management? | 1. Refer to colposcopy 2. Repeat test in 5 years 3. Repeat test in 1 year 4. Don’t know / Prefer not to say |
| What details should you provide the patient when you disclose the test results? | |
| - HPV is sexually transmitted virus | 1. Always 2. Most of the time 3. Sometimes 4. Never 5. Don’t need to communicate the result |
| - HPV is a virus that can cause cancer | 1. Always 2. Most of the time 3. Sometimes 4. Never 5. Don’t need to communicate the result |
| - Vaccine protects against high-risk types but not all | 1. Always 2. Most of the time 3. Sometimes 4. Never 5. Don’t need to communicate the result |
| - Infection is usually transient and clears without treatment | 1. Always 2. Most of the time 3. Sometimes 4. Never 5. Don’t need to communicate the result |
| Vignette 2 |  |
| 35-year-old patient, not vaccinated against HPV, with no relevant medical history, smoker (10-20 cigarettes/day). Invited to perform cervical cancer screening with HPV test during the family planning consultation. The test is "positive for HPV 16". | |
| What is the correct management? | 1. Refer to colposcopy 2. Repeat test in 5 years 3. Repeat test in 1 year 4. Don’t know / Prefer not to say |
| What details should you provide the patient when you disclose the test results? | |
| - HPV is sexually transmitted virus | 1. Always 2. Most of the time 3. Sometimes 4. Never 5. Don’t need to communicate the result |
| - HPV is a virus that can cause cancer | 1. Always 2. Most of the time 3. Sometimes 4. Never 5. Don’t need to communicate the result |
| - The infection can be transient but should not be underestimated because It has a higher risk of cancer | 1. Always 2. Most of the time 3. Sometimes 4. Never 5. Don’t need to communicate the result |
| - Smoking is associated with a higher risk of progression to precancerous lesions and cancer | 1. Always 2. Most of the time 3. Sometimes 4. Never 5. Don’t need to communicate the result |
| Vignette 3 |  |
| 54-year-old postmenopausal patient, hypertensive and with high cholesterol levels, not vaccinated against HPV. She is frequently screened for cervical cancer and is currently in the sixth round of screening. Invited to perform cervical cancer screening with an HPV test in routine surveillance consultation. The test is “positive for high-risk HPV of types other than 16 and 18”. Due to this result, reflex cytology was performed, the result of which was “squamous changes of undetermined significance" - ASC-US". | |
| What is the correct management? | 1. Refer to colposcopy 2. Repeat test in 5 years 3. Repeat test in 1 year 4. Don’t know / Prefer not to say |
| After receiving the result, the patient asks why the test was changed, since she has always had cytology. She fears that she may have cancer. She feels ashamed and angry because she thinks that her husband has been unfaithful to her. Still, she doesn't believe the test results because she doesn't have any symptoms of an infection. | |
| In response to these questions, the patient is informed that: | |
| - HPV testing is currently the recommended screening test because it is more sensitive than cytology | 1. Always 2. Most of the time 3. Sometimes 4. Never |
| - Although some types of HPV can cause cancer, most infections are transient and do not progress to serious lesions or cancer | 1. Always 2. Most of the time 3. Sometimes 4. Never |
| - The infection may persist for long periods, making it impossible to determine when she was infected | 1. Always 2. Most of the time 3. Sometimes 4. Never |
| - HPV infection is usually asymptomatic. Hence the importance of screenings and regular clinical surveillance | 1. Always 2. Most of the time 3. Sometimes 4. Never |
|  | |
| Thank you for your participation.  Your collaboration was very important for our work.  Do you have a suggestion for improving the CCS? | |
|  | |

**Supplementary material 2**

**Supplementary Table 1:** Exploratory factor analysis of awareness and perceptions regarding human papillomavirus-based cervical cancer screening in Central Portugal, 2024

|  | **Factor 1**  **Perceived logistical barriers** | **Factor 2**  **Awareness of screening effectiveness** | **Factor 3**  **Perceived safety and reliability of human papillomavirus testing** |
| --- | --- | --- | --- |
| **Item** |  |  |  |
| The transition to human papillomavirus testing has led to more time spent on screening | 0.84 |  |  |
| The transition to human papillomavirus testing resulted in decreased patients’ compliance with the screening programme | 0.85 |  |  |
| The transition to human papillomavirus testing triggered excessive referral to hospital units | 0.72 |  |  |
| Cervical cancer screening helps to reduce mortality from cervical cancer |  | 0.88 |  |
| Cervical cancer screening allows the treatment of pre-invasive disease and prevents progression to cervical cancer |  | 0.89 |  |
| Human papillomavirus testing is more sensitive than cytology |  |  | 0.81 |
| With a negative human papillomavirus test, it is safe to repeat cervical cancer screening at five years |  |  | 0.85 |
| Explained variance | 27.8% | 22.9% | 20.3% |
| Eigenvalue | 2.18 | 1.62 | 1.18 |
| Internal Consistency (Cronbach’s α) | 0.71 | 0.75 | 0.59 |

**Extraction method:** principal component analysis; **rotation method**: Varimax with Kaiser normalization. **Total variance explained**: 71.1%.

***Supplementary* Table 2**: Correct response rates and overall knowledge scores for human papillomavirus infection, human papillomavirus testing and cervical cancer screening guidelines among healthcare professionals in Central Portugal, 2024.

|  | **Total**  **n = 470** | **General practitioners**  **n = 227** | **Nurses**  **n = 243** |
| --- | --- | --- | --- |
| Human papillomavirus infection | | | |
| Overall knowledge score, mean (SD), % | 88.8 (11.4) | 90.2 (10.7) | 87.3 (11.9) |
| Human papillomavirus infection is rare (False) | 408 (86.8%) | 194 (85.5%) | 214 (88.1%) |
| Human papillomavirus infection occurs only in individuals with multiple sexual partners (False) | 449 (95.5%) | 223 (98.0%) | 226 (93.0%) |
| Human papillomavirus is exclusively sexually transmitted (False) | 239 (50.9%) | 122 (53.7%) | 117 (48.1%) |
| Human papillomavirus infection can occur throughout life (True) | 448 (95.3%) | 220 (96.9%) | 228 (93.8%) |
| Human papillomavirus infection is usually asymptomatic (True) | 454 (96.6%) | 223 (98.2%) | 231 (95.1%) |
| Human papillomavirus infection may have been acquired at any time in the past, and it is not possible to determine when it was acquired (True) | 447 (95.1%) | 219 (96.5%) | 228 (93.8%) |
| Most sexually active people can get human papillomavirus at some point in their lives (True) | 436 (92.8%) | 215 (94.7%) | 221 (90.9%) |
| Persistent human papillomavirus infection is associated with the development of precancerous lesions and cancer (True) | 436 (92.8%) | 215 (94.7%) | 221 (90.9%) |
| Human papillomavirus testing | | | |
| Overall knowledge score, mean (SD), % | 67.8 (19.3) | 75.4 (15.9) | 60.6 (19.5) |
| Human papillomavirus testing effectively assesses cervical cancer risk (True) | 437 (93.0%) | 214 (94.3%) | 223 (91.8%) |
| A positive human papillomavirus test is always an indication for colposcopy (False) | 334 (71.1%) | 171 (75.3%) | 163 (67.1%) |
| A positive human papillomavirus test means that a woman has or will have cervical cancer (False) | 418 (89.0%) | 218 (96.0%) | 200 (82.3%) |
| A negative human papillomavirus test means a very low risk of cervical cancer (True) | 341 (72.6%) | 204 (89.5%) | 137 (56.6%) |
| Cervical cancer screening guidelines | | | |
| Overall knowledge score, mean (SD), % | 82.9 (16.9) | 86.1 (15.0) | 79.9 (18.0) |
| Recommended age to begin screening (25 years) | 452 (96.2%) | 214 (94.3%) | 238 (97.9%) |
| Recommended screening interval after a negative human papillomavirus test (5 years) | 450 (95.7%) | 222 (97.8%) | 228 (93.8%) |
| Management following screening results | | | |
| Clinical vignette 1 (repeat testing after one year) | 322 (68.5%) | 172 (75.8%) | 150 (61.7%) |
| Clinical vignette 2 (referral to a cervical pathology unit for further testing) | 406 (86.4%) | 208 (91.6%) | 198 (81.5%) |
| Clinical vignette 3 (referral to a cervical pathology unit for further testing) | 318 (67.7%) | 160 (70.5%) | 158 (65.0%) |

3. SD, standard deviation. Individual knowledge items are presented as n (%) of participants who answered correctly. Overall knowledge scores are presented as mean (SD) percentages

**Supplementary Table 3** : Awareness and perceptions regarding cervical cancer screening and human papillomavirus testing among general practitioners and nurses in Central Portugal, 2024.

| **Survey item** | **Overall (%)** | **General practitioners n=227, %** | **Nurses n=243, %** | ***p value* *** |
| --- | --- | --- | --- | --- |
| Cervical cancer screening helps reduce cervical cancer mortality | 98.7 | 97.4 | 100.0 | 0.06 |
| Cervical cancer screening allows treatment of pre-invasive disease and prevents progression to cervical cancer | 97.6 | 97.3 | 97.9 | 0.64 |
| Human papillomavirus testing is more sensitive than cytology | 75.7 | 82.0 | 70.0 | 0.02 |
| With a negative human papillomavirus test, it is safe to repeat cervical cancer screening after five years | 65.5 | 83.3 | 49.0 | <0.01 |

Results are presented as the percentage of participants who selected " Agree” or “Strongly Agree ". *p* values *were* obtained using Pearson’s χ2 test based on the full response distribution.

**Supplementary Table 4**: Perceived barriers related to the transition to primary human papillomavirus testing among healthcare professionals in Central Portugal, 2024

| **Survey item** | **Group** | **Agree/strongly agree, %** | **Disagree/strongly disagree, %** | ***p value**** |
| --- | --- | --- | --- | --- |
| The transition to human papillomavirus testing led to more time spent on screening | General practitioners | 4.8 | 78.4 | 0.01 |
|  | Nurses | 9.0 | 70.0 |  |
|  | Total | 7.0 | 74.0 |  |
| The transition to human papillomavirus testing decreased patient adherence to screening | General practitioners | 2.2 | 89.4 | 0.48 |
|  | Nurses | 2.1 | 86.4 |  |
|  | Total | 2.1 | 87.9 |  |
| The transition to human papillomavirus testing decreased healthcare professionals' adherence to screening | General practitioners | 0.0 | 90.7 | 0.06 |
|  | Nurses | 2.1 | 86.4 |  |
|  | Total | 1.1 | 88.5 |  |
| The transition to human papillomavirus testing triggered excessive referrals to hospital units | General practitioners | 11.4 | 58.6 | 0.35 |
|  | Nurses | 9.1 | 64.2 |  |
|  | Total | 10.2 | 61.5 |  |
| Delays in scheduling first appointments at colposcopy units are a barrier to effective screening | General practitioners | 39.6 | 38.8 | 0.02 |
|  | Nurses | 26.7 | 44.4 |  |
|  | Total | 33.0 | 41.7 |  |

*Pearson’s χ^2^ test based on the full response distribution.

**Supplementary Table 5:** Confidence in addressing human papillomavirus-related topics and frequency of communication among healthcare professionals in Central Portugal, 2024

|  | **General practitioners**  **(n=227),**  **Mean (SD)** | **Nurses**  **(n=243)**  **Mean (SD)** | ***p value*** |
| --- | --- | --- | --- |
| Confidence level in addressing human papillomavirus-related topics | | | |
| Cervical cancer | 4.33 (0.67) | 4.06 (0.87) | <0.01* |
| Human papillomavirus infection | 4.30 (0.65) | 3.92 (0.97) | <0.01* |
| Human papillomavirus-based cervical screening | 4.50 (0.54) | 4.16 (0.86) | <0.01* |
| Positive human papillomavirus test results | 4.22 (0.70) | 3.62 (1.11) | <0.01** |
| Communication of human papillomavirus test results | | | |
| Positive human papillomavirus test results | 4.32 (1.08) | 3.60 (1.46) | <0.01** |
| Negative human papillomavirus test results | 2.82 (1.27) | 2.82 (1.30) | 0.96* |
| Frequency of information communicated to patients in clinical vignettes | | | |
| First vignette |  |  |  |
| Human papillomavirus is a sexually transmitted virus | 1.94 (1.04) | 2.05 (1.04) | 0.24** |
| Human papillomavirus is a virus that can cause cancer | 1.66 (0.88) | 1.87 (1.05) | 0.01** |
| The vaccine protects against most high-risk human papillomavirus types, but not all | 4.32 (0.82) | 3.97 (1.25) | 0.01** |
| The infection is usually transient and clears without treatment, especially in young women | 3.93 (1.25) | 2.86 (1.23) | <0.01** |
| Second vignette |  |  |  |
| Human papillomavirus is a sexually transmitted virus | 1.80 (0.99) | 1.97 (1.15) | 0.09* |
| Human papillomavirus is a virus that can cause cancer | 1.57 (0.79) | 1.74 (0.98) | 0.01** |
| The infection can be transient but should not be underestimated | 4.28 (0.95) | 3.61 (1.55) | <0.01** |
| Smoking is associated with a higher risk of progression to precancerous lesions and cancer | 3.88 (1.30) | 3.36 (1.60) | <0.01** |
| Third vignette |  |  |  |
| Human papillomavirus testing the recommended screening test because it is more sensitive than the cytology | 4.35 (1.04) | 3.91 (1.56) | 0.01** |
| Some types of human papillomavirus can cause cancer, but most infections are transient | 4.13 (1.02) | 3.57 (1.45) | <0.01** |
| The infection may persist for long periods | 4.23 (1.12) | 3.67 (1.50) | <0.01** |
| Human papillomavirus infection is usually asymptomatic. Hence the importance of screenings and regular clinical surveillance | 4.61 (0.73) | 4.27 (1.27) | 0.01** |

* Independent-samples t-test; **Mann-Whitney-U test; Scores range from 1 to 5, with higher scores indicating greater confidence or more frequent communication. Items were reverse-coded as appropriate to ensure consistent score direction.

***Supplementary* Table 6:** Highest response categories for confidence and communication practices among healthcare professionals in Central Portugal, 2024

| **Topic** | **Response category** | **Total (n=470)**  **%** | **General Practitioners (n=227)**  **%** | **Nurses (n=243)**  **%** | ***p value* *** |
| --- | --- | --- | --- | --- | --- |
| Confidence in addressing human papillomavirus-related topics | | | | | |
| Cervical cancer | Confident /very confident | 92.1% | 94.7% | 89.7% | <0.01 |
| Human papillomavirus infection | Confident /very confident | 93.0% | 96.7% | 83.5% | <0.01 |
| Human papillomavirus -based cervical screening | Confident /very confident | 95.1% | 98.7% | 91.8% | <0.01 |
| Positive human papillomavirus test results | Confident /very confident | 84.3% | 93.8% | 75.9% | <0.01 |
| Frequency of communication of human papillomavirus test results | | | | | |
| Positive human papillomavirus result | Always | 50.0% | 62.6% | 38.3% | <0.01 |
| Negative human papillomavirus result | Always | 13.8% | 15.0% | 12.8% | 0.38 |
| Frequency of communication of human papillomavirus infection and screening-related information | | | | | |
| The vaccine protects against most high-risk human papillomavirus types | Always | 44.3% | 49.8% | 39.1% | <0.01 |
| Human papillomavirus testing is more sensitive than cytology | Always | 52.6% | 57.7% | 47.7% | 0.01 |
| Human papillomavirus infection usually clears spontaneously | Always | 26.8% | 41.0% | 13.6% | <0.01 |
| Human papillomavirus infection can be transient but should not be underestimated | Always | 41.3% | 52.4% | 30.9% | <0.01 |
| Smoking increases the risk of cervical cancer | Always | 33.8% | 42.7% | 25.5% | <0.01 |
| Most human papillomavirus infections do not progress to cancer | Always | 32.1% | 40.5% | 24.3% | <0.01 |
| Human papillomavirus infection may persist for long periods | Always | 39.6% | 51.1% | 28.8% | <0.01 |
| Human papillomavirus infection is usually asymptomatic | Always | 62.8% | 69.2% | 56.8% | <0.01 |
| Frequency of communication of human papillomavirus transmission and oncogenic risk | | | | | |
| Human papillomavirus is a sexually transmitted virus (Vignette 1) | Always | 33.0% | 40.1% | 26.3% | <0.01 |
| Human papillomavirus is a sexually transmitted virus (Vignette 2) | Always | 37.0% | 47.1% | 27.6% | <0.01 |
| Human papillomavirus can cause cancer (Vignette 1) | Always | 41.3% | 53.3% | 30.0% | <0.01 |
| Human papillomavirus can cause cancer (Vignette 2) | Always | 45.5% | 58.1% | 33.7% | <0.01 |

Results are presented as the percentage of participants selecting the highest response category within each item ("Confident/Very confident" or "Always"), as appropriate. *Pearson’s χ2 test based on the full response distribution.
